# Transport and Point Contact Measurements on Pr_1−x_Ce_x_Pt_4_Ge_12_ Superconducting Polycrystals

**DOI:** 10.3390/nano10091810

**Published:** 2020-09-10

**Authors:** Paola Romano, Francesco Avitabile, Angela Nigro, Gaia Grimaldi, Antonio Leo, Lei Shu, Jian Zhang, Antonio Di Bartolomeo, Filippo Giubileo

**Affiliations:** 1 Science and Technology Department, Via De Sanctis, University of Sannio, I-82100 Benevento, Italy; promano@unisannio.it (P.R.); favitabile@unisa.it (F.A.); 2 CNR-SPIN Salerno, via Giovanni Paolo II n. 132, 84084 Fisciano, Italy; gaia.grimaldi@spin.cnr.it (G.G.); adibartolomeo@unisa.it (A.D.B.); 3 Physics Department “E. R. Caianiello”, University of Salerno, via Giovanni Paolo II n. 132, 84084 Fisciano, Italy; nigro@sa.infn.it (A.N.); antonio.leo@fisica.unisa.it (A.L.); 4 NANO_MATES Research Center, Università degli Studi di Salerno, I-84084 Fisciano (SA), Italy; 5 State Key Laboratory of Surface Physics, Department of Physics, Fudan University, Shanghai 200433, China; leishu@fudan.edu.cn (L.S.); 14110190050@fudan.edu.cn (J.Z.); 6 Shanghai Research Center for Quantum Sciences, Shanghai 201315, China

**Keywords:** superconductivity, transport properties, energy gap, superconducting order parameter, proximity effect, nano-junction, Andreev reflection

## Abstract

We performed a detailed investigation of the superconducting properties of polycrystalline Pr_1−x_Ce_x_Pt_4_Ge_12_ pellets. We report the effect of Ce substitution, for x = 0.07, on magnetic field phase diagram H-T. We demonstrate that the upper critical field is well described by the Ginzburg–Landau model and that the irreversibility field line has a scaling behaviour similar to cuprates. We also show that for magnetic fields lower than 0.4 T, the activation energy follows a power law of the type 𝐻^−1/2^, suggesting a collective pinning regime with a quasi-2D character for the Ce-doped compound with x = 0.07. Furthermore, by means of a point contact Andreev reflection spectroscopy setup, we formed metal/superconductor nano-junctions as small as tens of nanometers on the PrPt_4_Ge_12_ parent compound (x = 0). Experimental results showed a wide variety of conductance features appearing in the dI/dV vs. V spectra, all explained in terms of a modified Blonder–Tinkham–Klapwijk model considering a superconducting order parameter with nodal directions as well as sign change in the momentum space for the sample with x = 0. The numerical simulations of the conductance spectra also demonstrate that s-wave pairing and anisotropic s-waves are unsuitable for reproducing experimental data obtained at low temperature on the un-doped compound. Interestingly, we show that the polycrystalline nature of the superconducting PrPt_4_Ge_12_ sample can favour the formation of an inter-grain Josephson junction in series with the point contact junction in this kind of experiments.

## 1. Introduction

Filled skutterudite materials have attracted a great deal of attention for a large number of properties such as metal–insulator transitions, spin fluctuations, and heavy fermion behaviour [1,2,3,4]. Several compounds in the family of filled skutterudites also show the phenomenon of superconductivity [5,6,7,8]. They have the chemical formula MT_4_X_12_, where M is an electropositive metal (Sr, Ba, La, Pr, Th), T is a transition metal (Fe, Os, or Ru), and X usually represents a pnictogen (Sb, As, or P). The first Pr-based superconductor to be discovered was the heavy-fermion PrOs_4_Sb_12_, with a critical temperature T_c_ = 1.85 K, showing intriguing properties such as a giant electronic specific heat coefficient [1]. Moreover, experiments of thermal transport [9] on single crystals evidenced the possible existence of a superconducting phase at high magnetic fields in which the energy gap has at least four point nodes, and a second phase at low magnetic fields in which the energy gap is characterized by only two point nodes. Recently, a new Pt-based family of skutterudite, with chemical formula MPt_4_Ge_12_, was synthetized, showing superconducting properties at relatively high temperatures. In particular, the compound with praseodymium (Pr) as metal shows a transition temperature T_c_ = 7.9K, while for the compound with Lanthanum (M = La), T_c_ = 8.3 K has been reported [10], as confirmed by electrical resistivity, magnetic susceptibility and specific heat measurements. Nuclear magnetic resonance experiments have given indications for conventional superconductivity in LaPt_4_Ge_12_ [11].

Lower superconducting critical temperature was previously reported for other MPt_4_Ge_12_ compounds such as SrPt_4_Ge_12_ (T_c_ = 5.10 K) and BaPt_4_Ge_12_ (T_c_ = 5.35 K) [12]. The higher critical temperatures for Pr and La compounds with respect to Sr and Ba compounds have been explained as the existence of a larger density of states at the Fermi level, as resulting from ^73^Ge nuclear quadrupole resonance experiments at zero field [13]. It has also been suggested that PrPt_4_Ge_12_ and LaPt_4_Ge_12_ can be characterized by two superconducting gaps. Indeed, according to heat capacity measurements as a function of temperature and magnetic field, the superconducting state cannot be explained by considering a single isotropic or anisotropic energy gap [14]. The presence of two distinct linear regions in the magnetic field dependence of the Sommerfeld coefficient of electronic heat capacity was interpreted as a possible indication for two-gap superconductivity in these compounds. The critical current density and pinning force of superconducting PrPt_4_Ge_12_ was measured in magnetization experiments [15], revealing that dependence of both quantities with respect magnetic field can be explained using a double exponential model already developed to explain the properties of the two-band superconductor MgB_2_ [16,17,18,19].

μSR experiments on PrPt_4_Ge_12_ showed a time-reversal symmetry breaking below T_c_ [20,21,22,23], in contrast to the results on LaPt_4_Ge_12_, for which the time-reversal symmetry breaking is absent and a conventional superconductivity with a fully gapped density of states is supposed [21]. The superconducting order parameter of LaPt_4_Ge_12_ has also been studied using specific heat and thermal conductivity measurements [24], showing that the sharp transition in the specific heat and its zero-field temperature dependence are well described in a conventional BCS (Bardeen–Cooper–Schrieffer) scenario characterized by a single energy gap and s-wave symmetry [25]. On the other hand, de Haas–van Alphen measurements have been reported with state-of-the-art band-structure calculations showing that LaPt_4_Ge_12_ and PrPt_4_Ge_12_ have almost identical electronic structures, Fermi surfaces and effective masses [26]. So far, few investigations have been reported that probe the superconducting energy gap in the PrPt_4_Ge_12_ compound, with results supporting both nodal and nodeless energy gaps. Recently, electrical resistivity, magnetic susceptibility, specific heat, and thermoelectric power experiments were performed on Pr_(1-x)_Ce_x_Pt_4_Ge_12_ to investigate the influence of the magnetic state of the Ce ions on the superconducting properties of the compound [27]. Interestingly, the results indicate a crossover from a nodal to a nodeless superconducting energy gap and that PrPt_4_Ge_12_ could be a two-band superconductor in which the electron scattering due to Ce substitution can suppress the superconductivity within one of the bands.

In this paper, we perform a detailed study of the superconducting transport properties of the Pr_1−x_Ce_x_Pt_4_Ge_12_ compound for x = 0.07. We measured the resistive transitions for the sample with x = 0.07 in external applied magnetic fields. We deduced the H-T phase diagram of the Ce-doped sample Pr_0.93_Ce_0.07_Pt_4_Ge_12_, that is the upper critical field H_c2_, as well as the irreversibility line. We also analysed the resistive transition data in the framework of the thermally assisted motion of vortices. We also performed direct measurements of the superconducting energy gap in the parent compound PrPt_4_Ge_12_ (x = 0) by means of point contact spectroscopy experiments, which made it possible to realize metal/superconductor nano-junctions with dimensions of few nanometres. We measured the conductance spectra of the point contact junction at low temperature (4.2 K) and we demonstrated that the conductance feature can be reproduced in a theoretical model that takes into account the symmetry of the superconducting order parameter with nodal directions and change of sign in the momentum space. We also estimated the superconducting energy gap for the sample PrPt_4_Ge_12_ in the range 0.55–0.95 meV.

## 2. Materials and Methods

Polycrystalline Pr_1−x_Ce_x_Pt_4_Ge_12_ pellets (with x = 0, 0.07, 0.1) were synthesized in argon atmosphere by arc melting, using Pr ingots, Ce rods, Pt sponge, and Ge pieces as preparation materials, weighed in stochiometric ratios. Arc melting and turning over were repeated five times to obtain high chemical homogeneity. The samples were then annealed in a sealed quartz tube in 200 Torr argon atmosphere at 800 °C for 14 days. X-Ray diffractometry (reported elsewhere [27]) confirmed the sample quality evidencing the expected cubic skutterudite crystal structure.

Measurements of sample resistance as a function of the temperature, *R*(*T*), were carried out by standard four-probe offset-compensated technique inside a Cryogenic Ltd. cryogen-free magnet system, equipped with a variable temperature insert (vertically inserted inside a superconducting solenoid for fields up to 9T) in which the temperature can vary in the range 1.6 K–325 K. A Keithley 2430 DC current source was employed to bias the sample, while measuring the voltage by means of a Keithley 2182 nanovoltmeter.

Point Contact Spectroscopy experiments were performed by means of a home-built mechanical inset equipped with a screw-driven chariot to move a metallic tip towards the surface of the superconducting sample, in order to form a metal/superconductor nanometric constriction, the so-called point contact junction. The junction is then cooled down by immersing the inset in a liquid helium cryostat to perform current-voltage (I–V) measurements at low temperature by standard four-probe technique. Differential conductance (dI/dV vs. V) spectra are then obtained performing numerical derivative of the I–V curves.

## 3. Results and Discussion

### 3.1. Transport Properties

The dynamic behaviour of Abrikosov vortices in type II superconductors determines the transport properties of superconducting samples. In particular, vortices are set in motion when the Lorentz force, due to the bias current, exceeds the strength of vortex pinning forces, causing a nonzero Ohmic resistance. The presence of pinning, caused by any spatial inhomogeneity of the material like impurities, point defects, grain boundaries, etc., allows superconducting material to sustain current without flux motion and dissipation, giving rise to a nonzero critical current density. Different phases can be recognized in the magnetic field phase diagram *H-T* depending on the relative strengths of the pinning potential, the Lorentz driving energy, and the elastic energy of the vortex lattice, thermal energy and dimensionality. The interplay between these interactions results in several phase separation lines in the *H-T* phase diagram [28].

In the following, we characterize the *H-T* phase diagram of the sample Pr_0.93_Ce_0.07_Pt_4_Ge_12_, the upper critical field H_c2_, and the irreversibility line, above which the critical current density becomes zero. Then, the resistive transitions in external applied magnetic fields are analysed in the framework of the thermally assisted motion of vortices.

#### 3.1.1. Magnetic Field Temperature Phase Diagram

**Figure 1:**
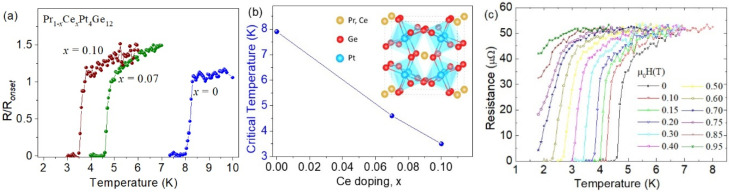
(**a**) The resistance as a function of the temperature normalized to the normal state resistance value for the three different Ce doping. (**b**) Evaluated critical temperature values as a function of the doping. The inset shows a schematic diagram of the Pr_1−x_Ce_x_Pt_4_Ge_12_ crystal structure, showing the Pr,Ce atoms residing in icosahedral cages formed by tilted PtGe_6_ octahedral. (**c**) Magnetic field dependence of the resistance versus temperature curve measured for the sample with x = 0.07 doping.

In Figure 1a we report the resistance vs. temperature curves, *R*(*T*), for Pr_1−x_Ce_x_Pt_4_Ge_12_ samples having x = 0, 0.07 and 0.10. The data were normalized to the normal state resistance, *R*_N_, evaluated just before the onset of the superconducting transition. We measured *R*_N_ = 4.3 mΩ for the undoped sample, *R*_N_ = 34 mΩ for sample with x = 0.07 and *R*_N_ = 63 mΩ for sample with x = 0.10. The superconducting critical temperature *T*_c_ was estimated at 50% of the onset transition resistance, obtaining *T*_c_ = 7.9 K for the undoped sample, *T*_c_ = 4.7 K for samples with x = 0.07, and *T*_c_ = 3.6 K for samples with x = 0.1. The evolution of the critical temperature *T*_c_ as a function of the Ce doping *x* is summarized in Figure 1b. The effect on T_c_ of the partial substitution of Pr by Nd has been also reported in samples with Nd content x_Nd_ up to 0.1 [29]. The critical temperature is weakly dependent by Nd content, being reduced by only 10% at x_Nd_ = 0.1. On the other hand, the effect of the Ce substitution is much more important; the T_c_ is reduced by 59% at x = 0.07 and by 45% at x = 0.1. The effect of externally applied magnetic field up to 1 T on the R(T) curve for sample with x = 0.07 is shown in Figure 1c.

**Figure 2:**
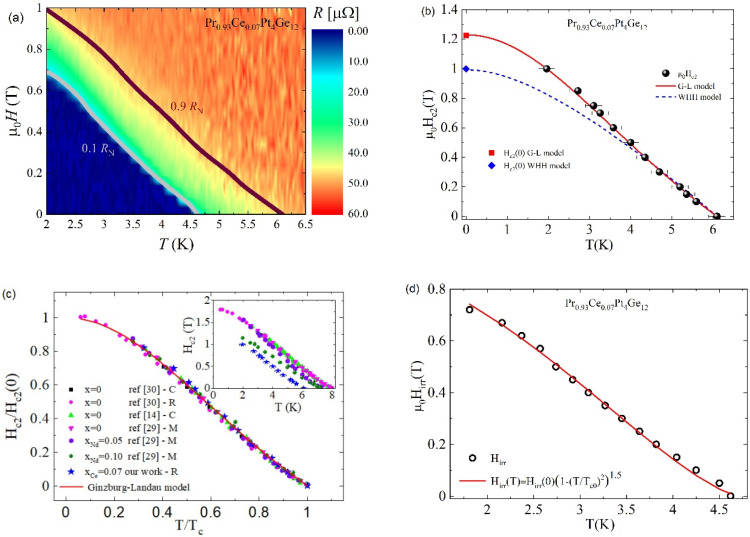
(**a**). The *H-T* phase diagram as obtained by R(T) measurements at different applied magnetic fields for the sample with the doping x = 0.07. The irreversibility line (0.1 R_N_ curve) and the upper critical field behaviour (0.9 R_N_ curve) are indicated. (**b**) The upper critical field $H_{c2}\left(T\right)$ data are shown as black spheres. The red solid line is a fit of the data by the Ginzburg–Landau Equation (Equation (1) in the text). The blue dashed line is obtained by the single band WHH model (Equation (2) in the text). (**c**) Scaling plots of the normalized upper critical field H_c2_(T)/H_c2_(0) plotted as function of the reduced temperature T/T_c_ for the parent compound PrPt_4_Ge_12_ [14,29,30], Nd-doped samples Pr_1−x_Nd_x_Pt_4_Ge_12_ [29] and for our Pr_1−x_Ce_x_Pt_4_Ge_12_ sample with x = 0.07. H_c2_(T) curves are obtained by resistivity, R, specific heat, C, and magnetization measurements, M. The inset shows the H_c2_ data as a function of the temperature for the same samples in the main panel. (**d**) The irreversibility field $H_{irr}\left(T\right)$ data are shown as open circles. The red solid line is a fit of the data by Equation (5) in the test.

In Figure 2a, the resulting magnetic field–temperature (*H-T*) phase diagram is shown, together with the upper critical field line *H*_c2_(T), which separates the normal and the superconducting state, and the irreversibility line $H_{irr}\left(T\right)$. In the *H-T* phase diagram, the $H_{irr}\left(T\right)$ line separates the Abrikosov vortex pinned regime and the vortex liquid regime, which is at a given temperature; the critical current density goes to zero at the irreversibility field. To evaluate the upper critical field, μ_0_*H*_c2_, we employed to the 90% of normal state resistance *R*_N_ criterion, while the irreversibility line, μ_0_*H*_irr_, was obtained using the 10% of *R*_N_ criterion. To analyse the temperature dependence of the upper critical field, the μ_0_*H*_c2_(*T*) data extracted by the *H-T* phase diagram are shown in Figure 2b.

The red solid line in Figure 2b represents the best fit of the data by the Ginzburg–Landau (G-L) formula:

(1) $$H_{c2}\left(T\right)=H_{c2}\left(0\right)\frac{\left(1-t^{2}\right)}{\left(1+t^{2}\right)}$$

where *H*_*c*2_(0) is the upper critical field at zero temperature and $t=T/T_{C}$ is the reduced temperature. The data are very well described by the G-L Equation with μ_0_*H*_*c*2_(0) = 1.23 T, as already reported for (Pr,La)Pt_4_Ge_12_ and Pr_1−x_Nd_x_Pt_4_Ge_12_ compounds [14,24,29,31,32,33,34]. For comparison, in Figure 2b, we also show the temperature dependence of the upper critical field derived within the Werthamer–Helfand–Hohenberg (WHH) model (blue dashed line), which includes orbital and Zeeman pair breaking [35,36]. In particular, for a single band superconductor in a dirty limit, the model yields:

(2) $$H_{c2}=\frac{2\Phi_{0}\;k_{B}\;T_{c}}{\hbar D_{0}}\;ht$$

where $\Phi_{0}$ is the magnetic flux quantum, $k_{B}$ the Boltzmann constant, $T_{c}$ the critical temperature in zero applied magnetic field, ħ is the reduced Planck constant, $D_{0}=\hbar /2m_{e}$ with $m_{e}$ the electron mass, and h is a parameter that runs from 0 to ∞ as T varies from $T_{c}$ to 0, while the reduced temperature $t=T/T_{c}$ is given by:

(3) $$\ln\left(t\right)=\Psi \left(\frac{1}{2}\right)-Re\Psi \left(\frac{1}{2}+h\left(i+d\right)\right)$$

where Ψ is the digamma function, $d=D/D_{0}$, and D the diffusivity of the band related to the slope of the μ_0_*H*_*c*2_(*T*) experimental data near $T_{c}$, given by $D=4k_{B}/\pi e\mu_{0}\left|dH_{c2}\left(T\right)/dT\right|$. For our sample, $\mu_{0}dH_{c2}\left(T\right)/dT=-0.23\;TK^{-1}$. Within this model, the zero-temperature upper critical field *H*_*c*2_(*0*) can be obtained by the well-known WHH formula:

(4) $$H_{c2}\left(0\right)=0.693\times T_{c}\left|\frac{dH_{c2}\left(T\right)}{dT}\right|$$

We note that the WHH model underestimates the $H_{c2}$ field at low temperatures, with the WHH formula giving μ_0_*H*_*c*2_(0) = 0.99 T, corresponding to the value experimentally measured at T = 2.1 K ($\mu_{0}H_{c2}=1\;T$).

The Ce substitution for Pr in PrPt_4_Ge_12_ does not seem to modify the behaviour of the temperature dependence of the upper critical field, showing a characteristic positive curvature near $T_{c}$, as for the parent compounds (Pr,La)Pt_4_Ge_12_ and the doped Pr_1−x_Nd_x_Pt_4_Ge_12_ material. To analyse the effects on the upper critical field H_c2_(T) curve of the partial substitution of Pr in PrPtGe in Figure 2c, we compare our findings for the sample with Ce doping x = 0.07 with the results reported in the literature for the un-doped parent compound PrPt_4_Ge_12_ [14,29,30] and for Nd-doped samples Pr_1−x_Nd_x_Pt_4_Ge_12_ [29]. A scaling behaviour described by the G-L formula, Equation (1), is obtained when the normalized upper critical field H_c2_(T)/H_c2_(0) is plotted as a function of the reduced temperature t = T/Tc. We also point out that the figure includes H_c2_(T) curves obtained by resistivity, specific heat, and magnetization measurements. Furthermore, the scaling behaviour is the same for the two different doping, Ce and Nd, despite the different strength of critical temperature lowering induced by the two type of doping.

Different mechanisms have been proposed to explain this behaviour, such as strong coupling effects, multi-band electronic structure, and disorder [37,38].

Moreover, the coherence length at zero temperature was evaluated by $\mu_{0}H_{c2}\left(0\right)=\Phi_{0}/4\pi \xi {\left(0\right)}^{2}$, with $\Phi_{0}$ being the magnetic flux quantum. A value of ξ(0)≈12 nm was obtained, which is close to the value reported for the un-doped compound, $\xi_{x=0}\left(0\right)\approx 14\;nm$ [39].

In Figure 2d, the temperature dependence of the irreversibility field is shown. The scaling relation,

(5) $$H_{irr}\left(T\right)=H_{irr}\left(0\right){\left(1-{\left(\frac{T}{T_{C0}}\right)}^{2}\right)}^{n}$$

was adapted to the experimental data with n=1.5 (red solid line in Figure 2c) and $\mu_{0}H_{irr}\left(0\right)=0.94T$. $T_{C0}$ is the zero field transition temperature, and the exponent n is determined by the flux pinning mechanism [40]. At temperatures close to $T_{C0}$, the scaling Equation was reduced to $H_{irr}\sim {\left(1-T/T_{C0}\right)}^{1.5}$ as observed by Yeshumn et al. for single crystals of YBa_2_Cu_3_O_7_ high-temperature superconductor and interpreted within the thermally activated flux-creep theory [41]. The exponent n = 1.5 is also consistent with the values found for the iron-based 122 and 1111-families and the TlSr_2_Ca_2_Cu_3_O_y_ compound [42,43,44].

In the field range between $H_{irr}\left(T\right)$ and $H_{c2}\left(T\right)$, the thermal fluctuations become important, and the superconducting state loses its zero-resistance behaviour. In type II superconductors, high-field and -current applications are limited by the irreversibility line $H_{irr}\left(T\right)$; for example, values of $H_{irr}$ at low temperatures from 50% to ∼80% of the $H_{c2}$ have been observed in MgB_2_ and up to 85% in (Y_0.77_,Gd_0.23_)Ba_2_Cu_3_O_y_ films [45,46]. In our sample, the irreversibility field was 70% of the upper critical field at low temperatures, and dropped to less than 20% of $H_{c2}$ close to the critical temperature $T_{C0}$.

#### 3.1.2. Temperature Dependence of the Vortex Activation Energy

**Figure 3:**
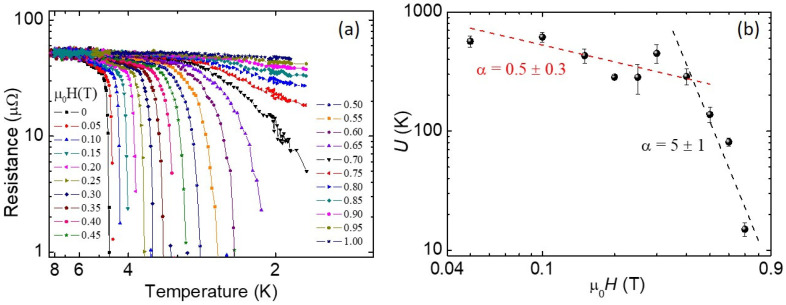
(**a**) The Arrhenius plot of the R(T) curves for the sample with the doping x = 0.07. (**b**) The pinning activation energy as a function of the applied magnetic field for the same sample. The dotted lines are obtained by a linear fit on the data in the log–log plot.

To further analyse the pinning properties of Pr_1−x_Ce_x_Pt_4_Ge_12_, we analysed the field dependence of the pinning activation energy *U*. This physical parameter was evaluated by a linear fit of the transition region in the *R*(*T*) curves represented in an Arrhenius plot, which are shown in Figure 3a. Indeed, the Arrhenius plots show that the resistivity is thermally activated over about two orders of magnitude at low fields; in this regime, the resistance’s dependence on temperature and field can be written in the form: $R\left(T,H\right)=R_{0}e^{-U\left(T,H\right)/k_{B}T}$ [47].

In Figure 3b, the resulting *U*(*H*) curve is shown. In particular, small activation energies, up to 600 K, are reported, close to the values $\sim 10^{3}\;K$ observed in Bi2212 superconductor [48]. According to the literature [34,36], the dependence of the pinning activation energy on the applied magnetic field follows a power law of the type $H^{-\alpha }$, with the exponent α, which assumes different values depending on the vortex pinning regime. In our case it is evident a crossover between two power law behaviours with different exponents. This crossover is at about 0.4 T, and the exponents are α ≈ 0.5 for μ_0_*H* < 0.4 T and α ≈ 5 for μ_0_*H* > 0.4 T. Both values can be associated with a collective pinning regime, with an exponent value between 0.5 and 1, which is usually found in cuprate superconductors and could be related to the quasi-2D character of these materials [41,49,50]. The existence of a crossover suggests the presence of two different pinning centres with different dimensions within a collective pinning regime, as was observed, for example, in undoped and Nd-doped PrPt_4_Ge_12_ samples [29], as well as YBa_2_Cu_3_O_7_ compounds [50] and in Nd_2−x_Ce_x_CuO_4−δ_ thin films [51].

### 3.2. Point Contact Spectroscopy

Point contact Andreev Reflection spectroscopy (PCAR) is a very powerful technique, widely applied to investigate the fundamental properties of superconductors, such as the superconducting energy gap amplitude, the density of states (DOS) at the Fermi level, and the symmetry of the superconducting order parameter (OP) [52]. PCAR experiments have been reported to study conventional BCS superconductors [53], high Tc cuprates (both hole doped and electron doped) [54,55,56,57,58,59], multiband superconductors [60,61,62,63,64], ruthenocuprates [65], iron-pnicniteds [66], heavy fermion superconductors [67], non-centrosymmetric superconductors [68], and topological superconductors [69]. This technique has also been successfully applied for precise measurements of the thickness and of the polarization in thin ferromagnetic/superconductor multilayers [70,71,72,73,74,75]. The PCAR technique consists of realizing a nano-contact between a tip-shaped normal-metal (N) electrode and a superconductor (S), thus forming a N/S nano-junction. By tuning the transparency of the N/S interface (i.e., by changing the tip pressure on the sample surface) one can realize different tunnelling regimes, going from the Andreev reflection [76,77] regime (in the case of a low potential barrier at the interface, corresponding to high interface transparency) to quasiparticle tunnelling regime for low interface transparency (high potential barrier). In a typical PCAR experiment, an intermediate regime can be achieved, with both Andreev reflection processes and quasiparticle tunnelling contributing to current transport through the N/S interface. If an electron travels from the normal side of the junction, with an energy lower than the superconducting energy gap, towards the N/S interface, it can enter into the superconducting side only as a Cooper pair, i.e., forming a pair with another electron, while originating a reflected hole in N with the opposite momentum. Consequently, a single Andreev reflection event causes a charge transfer to the S side of 2*e*, with *e* the electron charge. From a theoretical point of view, the transport through a point contact junction between a normal metal and a conventional BCS superconductor (with isotropic s-wave symmetry of the superconducting OP) was described in the BTK theory [78], in which the interface barrier is modelled by a dimensionless parameter Z. The case Z = 0 corresponds to an N/S junction, with a completely transparent barrier, in which the Andreev process is the dominant mechanism responsible for the transport current. On the other hand, Z > 1 represents a junction with a low transparent barrier, corresponding to a dominant tunnelling current flowing through the junction.

The BTK theory was subsequently extended to the case of superconductors with amplitude variation of the OP in the k-space (as for an anisotropic s-wave) and to the case of unconventional superconductors, in which the sign of the OP may also change (as for d-wave symmetry) [79]. It has been demonstrated that if an incident quasiparticle at the N/S interface experiences a different sign of the OP, Andreev bound states are formed at the Fermi energy [80]. From an experimental point of view, the formation of Andreev bound states are seen in the differential conductance spectra as a peak at zero bias [81,82,83,84,85]. In the case of d-wave symmetry, conductance depends on both the incident angle *ϕ* of the quasiparticle at the N/S interface and on the orientation angle *α* between the *a-*axis of the superconducting order parameter and the crystallographic axis. The conductance expression can be reduced to the model for anisotropic s-wave by simply assuming costant phase and assuming that only the OP amplitude changes in the *k*-space. We mention here that the BTK model and its extended version applied here do not consider the possible effects due to the case of energy-dependent DOS (the effects being mostly expected on the thermoelectric effects in NS junctions [86].

#### PCAR Experiment

The PCAR experiment was performed on the Pr_1−x_Ce_x_Pt_4_Ge_12_ sample having x = 0 (T_c_ = 7.9 K). We used a gold tip as a normal metal electrode, which was gently pushed onto the sample surface to realize the N/S nanoconstriction. The setup was then immersed in a helium liquid bath for low-temperature (T = 4.2 K) characterization. We measured the current–voltage characteristics I–V using a standard four-probe configuration, using a dc current supply to bias the junction and measuring the voltage by a nano-voltmeter. The conductance curves, d*I*/d*V–V,* were obtained by numerical derivation of the I–V curves. The PCAR setup also makes it possible to vary the tip pressure on the sample, obtaining a tuning of the barrier transparency and, consequently, different junction resistances. In our experiment, we obtained junction resistances R_N_ in the range 0.1 Ω−50 Ω. We noticed here that we did not have a direct control of the geometrical dimensions of the N/S junction formed in the PCAR experiment. However, we estimated the junction size through the Sharvin formula $R_{N}=4\rho \ell /\left(3\pi d^{2}\right)$, in which the normal resistance of the junction is related to the resistivity ρ = 3.5 μΩcm [87] and the mean free path ℓ = 103 nm [87] in the superconducting material, as well as to contact dimension d.

**Figure 4:**
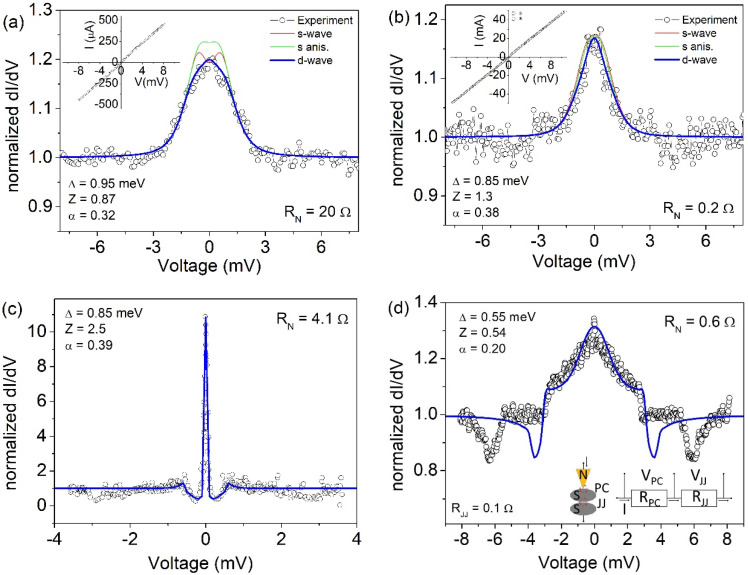
Normalized conductance spectra, dI/dV–V, measured at low temperature (T = 4.2 K) in different sample locations on the Pr_1−x_Ce_x_Pt_4_Ge_12_ (with x = 0) sample. Experimental data (empty symbols) in (**a**,**b**) are compared to numerically calculated curves for the three different symmetries. The I–V curve is shown in the inset. Experimental data (empty symbols) in (**c**,**d**) are compared to numerically calculated curves for d-wave symmetry only. Inset in (**d**) represent the schematic of model in which an inter-grain Josephson junction is formed in series with the point contact junction.

In Figure 4, we report normalized d*I*/d*V–V* curves, measured at low temperature (T = 4.2 K) for different nano-junctions, realized by varying the tip pressure and/or position on the sample surface. The conductance curves in Figure 4a,b are characterized by a zero-bias conductance peak (ZBCP) and have normal resistance R_N_ of 20 Ω and 0.2 Ω, respectively. Based on the Sharvin formula, we found the junction size to be d = 7 nm and d = 65 nm, respectively. In both cases, this confirms that the point contact is in the ballistic regime [52], in which the size of the junction is smaller than the mean free path in the superconductor (d<<ℓ). This corresponds to the physical conditions for which an electron can accelerate freely through the point contact, with no heat generated in the contact region, allowing energy-resolved spectroscopy.

The ZBCPs reported in Figure 4a,b have similar height (~1.2) and energy width (~±3 meV), while presenting a quite different shape. Experimental data are compared to theoretical fittings obtained for the three mentioned symmetries of the OP. We notice that s-symmetries are not able to completely reproduce the observed features. This discrepancy is more evident in Figure 4a, where the blue solid line represents the simulation obtained assuming a d-wave symmetry, with Δ = 0.95 meV, Z = 0.87 and α = 0.32 as fitting parameters. The Z value gives an indication of an intermediate regime, in which both Andreev reflection and quasiparticle tunnelling contribute to the conduction mechanism. The α value is an indication that the current direction is in between the nodal direction (*α* = *π*/4) and *α* = 0 (corresponding to maximum energy gap amplitude). In Figure 4b, very similar fitting curves are obtained for the different symmetries, although in this case, too, the d-wave symmetry seems to better reproduce the experimental behaviour, with fitting parameters Δ = 0.85 meV, Z = 1.3 and α = 0.38.

**Figure 5:**
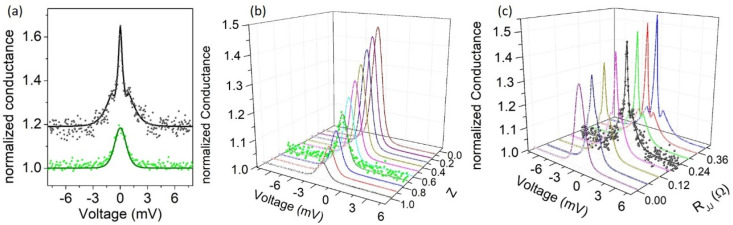
(**a**) Conductance spectra measured in a different location on the same superconducting sample: the lower (green) spectrum was measured soon after the tip approach on the surface. The upper spectrum was measured after increasing the tip pressure on the surface. The upper (black) spectrum was vertically shifted (+0.2) for clarity. Solid lines represent the numerical fits. (**b**) Evolution of conductance spectra (solid lines) calculated numerically for Δ = 0.55 meV and α = 0.46, and for 0 < Z < 1. The scattered (green) points refer to experimental data of Figure 5a. (**c**) Evolution of conductance spectra (solid lines) calculated numerically for Δ = 0.55 meV, Z = 0.39, α = 0.29, and 0 < $R_{JJ}$ < 0.42 Ω. The scattered (black) points refer to experimental data of Figure 5a.

On the other side, the spectra reported in Figure 4c,d appear very different. Indeed, in Figure 5c the ZBCP is very narrow and its amplitude is above 10, a value that cannot be obtained in s- or s-anisotropic fitting models, the maximum height being limited to 2. Moreover, at the side of the ZBCP, conductance minima are present, at voltages below ±1 mV. Such a feature is usually expected only when the superconducting OP is characterized by a sign change, as in the d-wave symmetry. Accordingly, we succeeded to simulate the experimental data by using the extended BTK model for a d-wave superconductor, assuming Δ = 0.85 meV, Z = 2.5 and α = 0.39. It was not possible to obtain similar features by applying the s-wave or s-anisotropic symmetry of the OP. We notice here that all experimental data have been simulated without introducing the so-called Γ-Dynes smearing factor [88] typically used to take into account possible pair-breaking effects of various origins (impurities, inelastic scattering, magnetic field, etc.).

Another completely different shape is observed in the spectrum of Figure 4d, where a wide ZBCP is followed by several features that cannot be reproduced by simply applying the model discussed above. However, we need to take into account that the superconducting sample is a pellet formed by pressed powders. Consequently, the tip pressure on the surface can cause the formation of an inter-grain Josephson junction in series with the point contact junction as depicted in the inset of Figure 4d. The effect of such inter-grain effects in point contact measurements has already been observed in experiments on MgB_2_ [61], MgCNi_3_ [89], and Pr_1−x_LaCe_x_CuO_4-y_ [57]. In this extended model, we need to take into account that the metallic tip (N electrode) forms a point contact junction on a superconducting grain, and this in turn forms a Josephson junction with another superconducting grain. Consequently, the total voltage drop *V* (experimentally measured) is given by the sum of the point contact *V_PC_* and the Josephson junction *V_JJ_* contributions $V=V_{PC}+V_{JJ}$. If the flowing current I is lower than $I_{JJ}$ there is no voltage drop at the inter-grain junction ($V_{JJ}=0$). Otherwise, *V_JJ_* can be calculated according to Lee formula [90] as $V_{JJ}=R_{JJ}I_{JJ}\sqrt{{\left(I/I_{JJ}\right)}^{2}-1}$, where $R_{JJ}$ and $I_{JJ}$ are the resistance and the critical current of the Josephson junction, respectively. If the flowing current I is lower than $I_{JJ}$ there is no voltage drop at the inter-grain junction ($V_{JJ}=0$). Then, the total conductance G can be calculated from the condition $1/G=\left[\frac{dV_{PC}}{dI}+\frac{dV_{JJ}}{dI}\right]$. We succeeded in simulating the conductance spectrum reported in Figure 4d assuming Δ = 0.55 meV, Z = 0.54 and α = 0.20. The fitting parameters related to the Josephson junctions were $R_{JJ}=$ 0.1 Ω and $I_{JJ}=$ 3.2 mA. We notice that these parameters are not completely free, being necessarily $R_{N}=R_{PC}+R_{JJ}$ and $R_{JJ}I_{JJ}<\Delta$. We remark here that neglecting the existence of a Josephson junction in series with the point contact would result in an over-estimation of the superconducting energy gap, because the measured voltage at which the conductance features are observed is larger than the real voltage $V_{PC}$ applied to the point contact junction ($V=V_{PC}+V_{JJ}$). We observe that the superconducting energy gap values obtained from the numerical fittings of most of the experimental spectra are in the range 0.85–0.95 meV that correspond to a ratio 2Δ/k_B_T_C_ in the range 2.5–2.8, smaller than the BCS value (3.52). In the case of the conductance curve of Figure 4d, we estimate a superconducting energy even smaller (Δ = 0.55 meV, i.e., 2Δ/k_B_T_C_ = 1.6). This may be an indication of suppressed superconductivity on the probed surface, with the point contact experiments being sensitive to a thin surface layer of tens of nanometres. Consequently, the correct procedure for estimating the ratio 2Δ/k_B_T_C_ would be to use the local critical temperature, which should be estimated based on the temperature evolution of the conductance spectra (not available in this experiment); this local Tc could be lower than the bulk sample Tc, giving an increased 2Δ/k_B_T_C_ ratio. We note that in the case of d-wave symmetry, electrons injected along different directions may experience different pairing amplitudes. Consequently, the shape of the conductance curves does not depend only on the height Z of the potential barrier at the interface, but also on the direction of the current injection. In Figure 5a, we show conductance measurements performed in a different location of the sample. The two spectra are the result of two successive measurements, in which the second spectrum was measured after increasing the tip pressure on the surface to increase the barrier transparency. The lower spectrum shows a ZBCP with limited amplitude (about 1.2) and is reproduced by the extended BTK model by assuming Δ = 0.55 meV, Z = 0.71 and α = 0.46. However, the second spectrum (shifted for clarity) has a much higher and more narrow ZBCP with two relative maxima appearing at the side of the peak. To understand the evolution of the second conductance curve, we show in Figure 5b the expected behaviour of the spectra obtained by keeping fixed the parameters Δ = 0.55 meV and α = 0.46, varying the barrier strength Z in the range 0 < Z < 1. We notice that in this scenario the main effect of the Z parameter is on the ZBCP height. The appearance of further conductance features is explained considering that the increased pressure of the tip on the surface has a double effect: it helps to obtain a more transparent barrier (lower Z), while it favours the formation of an inter-grain junction. Indeed, the numerical simulation of the experimental spectrum is obtained with good agreement by assuming Δ = 0.55 meV, Z = 0.39, α = 0.29 and $R_{JJ}=$ 0.22 Ω, $I_{JJ}=$ 0.24 mA. In Figure 5c, we show the evolution of conductance spectra numerically calculated by fixing the parameters Δ = 0.55 meV, Z = 0.39, α = 0.29 and varying only $R_{JJ}$ in the range 0–0.42 Ω.

## 4. Conclusions

We investigated the superconducting properties of polycrystalline Pr_1−x_Ce_x_Pt_4_Ge_12_ pellets, reporting the magnetic field phase diagram H-T for the Ce-doped compound with x = 0.07, as well as the point contact spectroscopy characterization of the parent compound PrPt_4_Ge_12_ (x = 0).

Interestingly, the irreversibility field line, found for Pr_0.93_Ce_0.07_Pt_4_Ge_12_, shows a scaling behaviour similar to high-temperature superconducting cuprates. The vortex activation energy was also evaluated at different applied magnetic fields. At magnetic fields lower than 0.4 T, the activation energy follows a power law of the type 𝐻^−α^, with the exponents α ≈ 0.5, which could indicate a collective pinning regime with a quasi-2D character.

For the compound with x = 0 (PrPt_4_Ge_12_), we realized normal metal/superconductor nano-junctions, with lateral dimensions of few nanometres, by pushing a gold tip onto the surface of polycrystalline sample. Several conductance spectra were measured at low temperatures, showing zero bias conductance peak with variable amplitude, height and width. All experimental data for the PrPt_4_Ge_12_ sample were consistently interpreted in the framework of extended BTK theory. A small energy gap was observed in the range 0.55 meV–0.95 meV, indicating the possible formation of inter-grain Josephson junctions in series with the point contact.
